# Association of bioimpedance‐derived parameters with pulse wave velocity: Phase angle, extracellular to intracellular water ratio, and body cell mass

**DOI:** 10.14814/phy2.70548

**Published:** 2025-09-10

**Authors:** Yujiro Asano, Motohiko Miyachi, Hinako Nanri, Haruka Murakami, Yuko Gando, Takashi Nakagata, Tsukasa Yoshida, Tomohiro Okura, Yosuke Yamada

**Affiliations:** ^1^ Doctoral Program in Physical Education, Health and Sport Science, Degree Programs in Comprehensive Human Sciences, Graduate School of Comprehensive Human Sciences University of Tsukuba Tsukuba Japan; ^2^ Center for Physical Activity Research National Institutes of Biomedical Innovation, Health and Nutrition Settsu Japan; ^3^ Department of Frailty Research, Center for Gerontology and Social Science National Center for Geriatrics and Gerontology Obu Japan; ^4^ Faculty of Sport Sciences Waseda University Saitama Japan; ^5^ Laboratory of Gut Microbiome for Health, Microbial Research Center for Health and Medicine National Institutes of Biomedical Innovation Ibaraki Japan; ^6^ Faculty of Sport and Health Science Ritsumeikan University Kusatsu Japan; ^7^ Faculty of Sports Science Surugadai University Hanno Japan; ^8^ Institute for Active Health Kyoto University of Advanced Science Kameoka Japan; ^9^ Institute of Health and Sport Sciences University of Tsukuba Tsukuba Japan; ^10^ R&D Center for Tailor‐Made QOL University of Tsukuba Tsukuba Japan; ^11^ Department of Sports and Health Sciences, Graduate School of Biomedical Engineering Tohoku University Sendai Japan; ^12^ Department of Medicine and Science in Sports and Exercise Tohoku University Sendai Japan

**Keywords:** arterial stiffness, bioelectrical impedance analysis, bioelectrical impedance spectroscopy, cardiovascular diseases, PWV

## Abstract

This study investigated the association between parameters derived from bioelectrical impedance spectroscopy (BIS) and arterial stiffness, as measured using carotid–femoral pulse wave velocity (cfPWV) and brachial‐ankle pulse wave velocity (baPWV) pulse wave velocities. Data from 292 Japanese adults were analyzed. BIS was used to assess the phase angle (PhA), extracellular water to intracellular water ratio (ECW/ICW), and body cell mass‐to‐free fat mass ratio (BCM/FFM). cfPWV and baPWV were measured using an Omron‐Colin device. Multiple linear regression analyses were conducted to examine the associations between BIS parameters and pulse wave velocity, adjusting for age, sex, height, smoking status, history of chronic diseases, and objectively measured physical activity. The results revealed that lower PhA, higher ECW/ICW, and lower BCM/FFM were significantly associated with higher cfPWV, independent of potential confounders. These associations remained significant even after adjusting for appendicular lean mass index. However, no significant associations were found between the BIS parameters and baPWV after controlling for covariates. These findings suggest that BIS‐derived parameters reflecting cellular health and body composition are associated with increased arterial stiffness in the central arteries, independent of muscle mass. This association may partly explain the relationship between these parameters and mortality, thereby highlighting the importance of BIS assessment in health promotion. However, future longitudinal studies are needed to confirm these findings.

## 1 INTRODUCTION

In clinical practice, bioelectrical impedance analysis (BIA) and bioelectrical impedance spectroscopy (BIS) are commonly used to evaluate body composition, particularly muscle mass and hydration. Both methods work in measuring two components: resistance (R) and reactance (Xc) (Baumgartner et al., 1988). Resistance represents a decrease in voltage, indicating conductivity through the ionic solutions of body water. In contrast, reactance refers to the delay in current flow, measured as a phase shift that reflects the capacitance of cell membranes and tissue interfaces (Jaffrin & Morel, 2008; Kyle et al., 2004; Norman et al., 2012). Recently, growing interest in qualitative changes in skeletal muscle exists, including functional and morphological aspects. These changes have been attributed to factors such as fat infiltration into the muscle, alterations in pennate angle, decreased motor neuron firing, shifts in muscle fiber composition, and changes in muscle cell volume (de Lucena Alves et al., 2023; Evans et al., 2019; Stock & Thompson, 2021; Wu et al., 2020). Recently, muscle quality parameters based on electrical properties assessed via BIA and BIS have gained attention. Among these, the phase angle (PhA) is a raw parameter that measures the phase difference (time delay) between the voltage and current at the cell membrane and tissue levels (Baumgartner et al., 1988; Lukaski & Talluri, 2023). In addition, water distribution parameters and their ratios, including extracellular water (ECW), intracellular water (ICW), and total body water, were also used to assess muscle quality (Hioka et al., 2021; Yamada, 2018; Yamada et al., 2023; Yoshida et al., 2018). At low frequencies, although only a small amount of the alternating current may penetrate the cell membrane, the majority primarily passes through the extracellular space. Conversely, at high frequencies, these currents can pass through the cell membranes and into the intracellular water (Yamada, 2018; Yoshida et al., 2018). Moreover, the body cell mass to free fat mass (BCM/FFM) ratio is an additional muscle quality parameter (Yamada et al., 2023). These muscle quality parameters have been reported to be associated with physical function, sarcopenia, disability, and subsequent mortality (Garlini et al., 2019; Iwasaka et al., 2023, 2024; Norman et al., 2023; Uemura et al., 2020; Wilhelm‐Leen et al., 2014; Yamada et al., 2021, 2023). Nevertheless, except for a reduction in physical impairment, the mechanisms underlying their association with mortality remain unclear.

Arterial stiffness and compliance can be assessed using pulse wave velocity (PWV), which is key to studying age‐related cardiovascular disease (CVD), as well as predicting the onset of hypertension, coronary artery disease, and cognitive decline (Budoff et al., 2022; Marshall et al., 2024). PWV is a valuable tool for assessing vascular aging and predicting CVD risk with greater accuracy than chronological age alone (Marshall et al., 2024; Vlachopoulos et al., 2019). Furthermore, PWV is associated with cardiovascular death and all‐cause mortality, independent of blood pressure (Vlachopoulos et al., 2019). Several studies have reported that arterial stiffening is associated with sarcopenia, reduced muscle mass and volume, and impaired physical function (Brunner et al., 2011; Piotrowicz et al., 2022; Rodríguez et al., 2017). However, most studies examining the relationship between PWV and sarcopenia and muscle tissue used brachial‐ankle PWV (baPWV) measurements obtained using a cuff‐based method, rather than the gold standard method of direct ECG‐gated carotid–femoral PWV (cfPWV) measurement (Laurent et al., 2006; Piotrowicz et al., 2022). Cuff‐based baPWV has been criticized for being strongly influenced by age and blood pressure levels. baPWV also combined multiple arterial segments into a single value, including large elastic arteries such as the descending aorta and stiffer peripheral arteries, that is, large conduit arteries (mainly the aorta) and muscular arteries (brachial and tibial) (Budoff et al., 2022; Lu et al., 2017; Piotrowicz et al., 2022; Yu et al., 2008). Therefore, the assessment of cfPWV is crucial for accurately evaluating arterial stiffness and compliance.

Muscle quality, including indicators such as PhA, ECW/ICW ratio, and BCM/FFM, can more precisely reflect the contractile component of muscle than muscle mass or volume alone. Although several studies have reported that lower muscle quality may be associated with CVD and mortality (Iwasaka et al., 2024; Knudsen et al., 2014; Langer et al., 2023; Raji et al., 2023), the relationship between muscle quality parameters, including PhA, ECW/ICW ratio, BCM/FFM, and PWV, remains unclear. Additionally, a paradox exists: Although moderate‐to‐vigorous intensity resistance training has been reported to improve muscle quality (Otsuka et al., 2022), some evidence suggests that this form of exercise may further increase PWV and arterial stiffness (Figueroa et al., 2019). Accordingly, in this study, we aimed to investigate the association between muscle quality (PhA, ECW/ICW ratio, BCM/FFM, cfPWV) and baPWV. We hypothesized that poorer muscle quality is associated with higher cfPWV, which reflects arterial stiffness. However, because baPWV is a variable that can be influenced by various factors, no significant associations are expected.

## 2 MATERIALS AND METHODS

### 2.1 Participants

This study was part of a longitudinal investigation conducted at the National Institute of Health and Nutrition in Tokyo (Tripette et al., 2021). Participants were selected based on the following criteria: (1) measurement of anthropometric variables, (2) assessment of physical activity using accelerometer‐based monitors, and (3) body composition measurements using BIS. Details of the recruitment process are described in a previous publication (Tripette et al., 2021). Participants with cardiovascular, respiratory, neurological, metabolic, or orthopedic disorders were excluded. The study was approved by the Institutional Review Board of the National Institute of Biomedical Innovation, Health and Nutrition (No. KENEI‐102). Written informed consent was obtained from all participants before their participation. All procedures adhered to the relevant guidelines and regulations.

### 2.2 Arterial stiffness

A vascular testing device (Form PWV/ABI; Omron Colin, Kyoto, Japan) was used to simultaneously measure baPWV, which reflects the central and peripheral arterial stiffness, and cfPWV, which reflects central arterial stiffness. Before the measurement, operators verified signal quality by confirming proper detection of blood pressure, electrocardiogram, and heartbeats on the device monitor. Pulse waveforms were analyzed using the device's built‐in automatic analysis algorithm. All measurements were conducted in the morning between 9:00 a.m. and 12:00 p.m., following a fasting period of at least 12 h. Although room temperature was not strictly controlled, the temperature remained stable within a range of approximately 20–25°C. Participants rested in the supine position for 10 min before the measurements were taken. All assessments were performed in this position. The reproducibility of PWV measurements has been confirmed in previous studies. In a preliminary reproducibility study conducted by trained examiners, the inter‐operator standard deviation was 51 cm/s for baPWV and 62 cm/s for cfPWV. Bilateral brachial and ankle arterial pressure waveforms were recorded for 10 s using extremity cuffs equipped with plethysmographic and oscillometric sensors on arms and ankles. baPWV was calculated by dividing the distance between the two arterial recording sites by the transit time. The mean values of both sides were used in the analysis. Moreover, carotid and femoral arterial pressure waveforms were recorded for 30 s using applanation tonometry sensors placed on the left common carotid and femoral arteries. cfPWV was determined by dividing the distance between the carotid and femoral artery sites by the pulse transit time.

### 2.3 Body composition

Body composition parameters, including appendicular lean mass, free fat mass, PhA, ECW/ICW ratio, and BCM/FFM, were evaluated using BIS (SFB7, ImpediMed, Pinkenba, Australia). The basic theory of BIS has been discussed in previous literature (Bartok & Schoeller, 2004; Sato et al., 2020; Yamada et al., 2010, 2017). Briefly, bioelectrical impedance was measured at 256 logarithmically distributed frequencies from 4 to 1000 kHz using disposable tab‐type monitoring electrodes (3 M Red Dot). The appendicular lean mass index (ALMI) was calculated as appendicular lean mass (estimated using the equations by Yamada, Nishizawa, et al., 2017), in kg divided by the height in m^2^ (kg/m^2^). PhA was calculated as the arctangent of reactance [Xc] divided by resistance (R), multiplied by 180°/*π*, at a frequency of 50 kHz. ECW, ICW, and FFM were estimated using equations developed by de Lorenzo et al. (De Lorenzo et al., 1997; De Lorenzo & Andreoli, 2003). The SFB7 system incorporates these equations (De Lorenzo et al., 1997; De Lorenzo & Andreoli, 2003). Resistance values at zero (*R*_0_) and infinite (*R*_*∞*_) frequencies were derived by fitting the bioimpedance spectra to the Cole–Cole model using ImpediMed's proprietary software. *R*_0_ corresponds to the resistance of ECW, whereas the resistance of ICW was estimated using the formula: 1/[(1/*R*_*∞*_) − (1/*R*_0_)]. The volumes of ECW and ICW were then calculated using the equations: ECW = (ρECW × Height)/*R*_0_ and ICW = (ρICW × Height)/Ri, where ρECW and ρICW represent the resistivity values for the extracellular (47 Ω·cm) and intracellular (273.9 Ω·cm) compartments, respectively. The ECW/ICW ratio was subsequently determined. BCM/FFM was calculated using the equations proposed by Wang et al.: BCM/FFM = 1.429/(1.569 + 1.16 × (ECW/ICW)) (Wang et al., 2005). Appendicular lean mass (ALM) was calculated using formulas established in a previous publication (Yamada, Nishizawa, et al., 2017). For men, the equation was ALM = (0.6947 × (height^2^/*Z*_50_)) − (55.24 × (*Z*_250_/*Z*_50_)) − (10,940 × (1/*Z*_50_)) + 51.33. For women, ALM = (0.6144 × (height^2^/*Z*_50_)) − (36.61 × (*Z*_250_/*Z*_50_)) − (9332 × (1/*Z*_50_)) + 37.91. The ALMI was then calculated by normalizing ALM to the square of the height (kg/m^2^). The between‐day reproducibility of bioimpedance‐derived parameters was assessed in our laboratory. The coefficients of variation for ECW and ICW measurements were 2.0% and 3.4%, respectively, whereas the corresponding intraclass correlation coefficients were 0.969 and 0.896, respectively.

### 2.4 Covariate measurements

All potential covariates were selected based on clinical findings and previous studies. These included age, sex, height, objectively measured physical activity level, smoking status, and history of hypertension, CVD, diabetes, dyslipidemia, and renal failure. Data on age, sex (male/female), renal failure (yes/no), and smoking status (never/quit smoking/current smoker) were obtained through self‐reported questionnaires. A history of dyslipidemia was defined as triglycerides ≥150 mg/dL or high‐density lipoprotein (HDL)‐cholesterol <40 mg/dL or being treated for this condition. The history of diabetes was defined as fasting glucose ≥110 mg/dL or being treated for diabetes. The history of hypertension was defined as systolic blood pressure ≥130 mmHg or diastolic blood pressure ≥85 mmHg, or being treated for hypertension. The history of CVD covered ischemic heart disease, cerebrovascular disease, and other heart conditions determined via self‐reported questionnaires. The presence of any of these diseases was classified as a history of CVD. Physical activity was assessed using a triaxial accelerometer (Actimarker EW4800; Panasonic, Osaka, Japan). The methodology and theoretical background are documented in previous studies (Yamada et al., 2018). Participants were instructed to wear the accelerometer for 20 days, with data collected over a 14‐day period during which the device was worn continuously from waking to bedtime. The device recorded metabolic equivalent tasks (MET) at 1‐min intervals. Physical activity level was quantified as the average METs multiplied by hours per day.

### 2.5 Statistical analysis

All statistical analyses were performed using R version 4.3.2 (R Foundation for Statistical Computing, Vienna, Austria). Statistical significance was set at *p* < 0.05. Multiple linear regression analyses were conducted to examine the independent associations of PhA, ECW/ICW ratio, and BCM/FFM with cfPWV and baPWV. Furthermore, we examined the association of ALMI with cfPWV and baPWV using the same modeling approach. Five models were constructed: a crude model with no covariates; model 1, adjusted for sex and age; model 2, model 1 plus height, smoking status, history of hypertension, cardiovascular diseases, diabetes, dyslipidemia, and renal failure; model 3, model 2 plus objectively measured physical activity level; and model 4, model 3 plus ALMI.

To enhance comparability and interpretability, we also included objectively measured physical activity level and ALMI as explanatory variables in the regression table, along with their corresponding summary statistics.

## 3 RESULTS

A total of 292 individuals were participated in this study. Participant characteristics are presented in Table 1. The mean age was 64.2 ± 11.3 years, with females comprising 79.8% of the sample.

**TABLE 1:** The characteristics of the study participants.

	Total
	*n* = 262
Female	233 (79.8)
Mean (SD), age, year	64.2 (11.3)
Mean (SD), height, cm	157.8 (8.0)
Mean (SD), weight, kg	56.7 (9.4)
Mean (SD), BMI, kg/m^2^	22.7 (3.1)
Smoking status	
Never	219 (75.0)
Quit	63 (21.6)
Current	10 (3.4)
Hypertension	134 (45.9)
Diabetes	23 (7.9)
Dyslipidemia	92 (31.5)
Cardiovascular diseases	37 (12.7)
Renal failure	2 (0.7)
Mean (SD), ALMI, kg/m^2^	7.2 (1.0)
Mean (SD), phase angle, degree	5.3 (0.8)
Mean (SD), ECF/ICF, ratio	0.78 (0.06)
Mean (SD), BCM/FFM, ratio	0.58 (0.02)
Mean (SD), cfPWV, cm/s	1030.0 (303.0)
Mean (SD), baPWV, cm/s	1423.7 (263.8)

*Note*: Values are presented as numbers (percentages) unless stated otherwise.

Abbreviations: ALMI, appendicular lean mass index; baPWV, brachial‐ankle pulse wave velocity; BCM/FFM, body cell mass to free fat mass ratio; BMI, body mass index; cfPWV, carotid–femoral pulse wave velocity; ECW/ICW, extracellular to intracellular water ratio.

Tables 2, 3, 4 present the results of multiple linear regression analyses examining the associations between ALMI and BIS‐derived muscle quality indicators (PhA, ECW/ICW ratio, and BCM/FFM) with cfPWV and baPWV.

**TABLE 2:** Associations of ALMI with carotid–femoral and brachial‐ankle pulse wave velocity (*n* = 292).

	Crude		Model 1		Model 2		Model 3	
	*β*	*p*‐Value	*β*	*p*‐Value	*β*	*p*‐Value	*β*	*p*‐Value
cfPWV								
ALMI	0.025	0.671	−0.084	0.181	−0.143	0.0216	−0.135	0.0294
Physical activity							−0.108	0.0269
baPWV								
ALMI	0.053	0.371	0.025	0.668	−0.087	0.109	−0.085	0.119
Physical activity							−0.029	0.505

*Note*: Crude model, no covariates; model 1, sex and age; model 2, model 1 + height, smoking status, history of hypertension, cardiovascular diseases, diabetes, dyslipidemia, and renal failure; model 3, model 2 + objectively measured physical activity level.

Abbreviations: ALMI, appendicular lean mass index; BCM/FFM, body cell mass‐to‐free fat mass ratio; ECW/ICW, extracellular‐to‐intracellular water ratio.

As shown in Table 2, higher ALMI was significantly associated with lower cfPWV after adjusting for sex, age, height, smoking status, and history of hypertension, cardiovascular disease, diabetes, dyslipidemia, and renal failure, as well as objectively measured physical activity level. However, no significant associations were observed between ALMI and baPWV in any of the models.

Table 3 shows that PhA, the ECW/ICW ratio, and the BCM/FFM ratio were significantly associated with cfPWV across all models. This includes the fully adjusted model (Model 4), which accounted for ALMI, objectively measured physical activity level, and other demographic and clinical covariates. Specifically, higher PhA and BCM/FFM, as well as a lower ECW/ICW ratio, were independently associated with lower cfPWV. These associations remained significant even after adjusting for ALMI and physical activity level.

**TABLE 3:** Associations of PhA, ECW/ICW ratio, and BCM/FFM with carotid–femoral pulse wave velocity (*n* = 292).

	Crude		Model 1		Model 2		Model 3		Model 4	
	*β*	*p*‐Value	*β*	*p*‐Value	*β*	*p*‐Value	*β*	*p*‐Value	*β*	*p*‐Value
Phase angle										
Phase angle	−0.343	<0.001	−0.229	0.0004	−0.196	0.0018	−0.179	0.0046	−0.158	0.0135
Physical activity							−0.094	0.0533	−0.092	0.0596
ALMI									−0.105	0.0927
ECW/ICW										
ECW/ICW	0.443	<0.001	0.207	0.0006	0.178	0.0021	0.164	0.0047	0.149	0.0108
Physical activity							−0.097	0.0461	−0.094	0.0541
ALMI									−0.112	0.0706
BCM/FFM										
ECW/ICW	−0.432	<0.001	−0.192	0.0013	−0.163	0.0044	−0.149	0.0093	−0.134	0.0204
Physical activity							−0.099	0.0432	−0.095	0.051
ALMI									−0.114	0.0667

*Note*: Crude model, no covariates; model 1, sex and age; model 2, model 1 + height, smoking status, history of hypertension, cardiovascular diseases, diabetes, dyslipidemia, and renal failure; model 3, model 2 + objectively measured physical activity level; model 4, model 3 + appendicular lean mass index.

Abbreviations: ALMI, appendicular lean mass index; BCM/FFM, body cell mass‐to‐free fat mass ratio; ECW/ICW, extracellular‐to‐intracellular water ratio; PhA, phase angle.

In contrast, as shown in Table 4, PhA, the ECW/ICW ratio, and BCM/FFM were not significantly associated with baPWV after adjusting for covariates in Models 1–4. Although significant associations were observed in the crude model, these associations were attenuated and became non‐significant after adjustment.

**TABLE 4:** Associations of PhA, ECW/ICW ratio, and BCM/FFM with brachial‐ankle pulse wave velocity (*n* = 292).

	Crude		Model 1		Model 2		Model 3		Model 4	
	*β*	*p*‐Value	*β*	*p*‐Value	*β*	*p*‐Value	*β*	*p*‐Value	*β*	*p*‐Value
Phase angle										
Phase angle	−0.32	<0.001	−0.047	0.433	−0.056	0.311	−0.051	0.362	−0.035	0.533
Physical activity							−0.027	0.534	−0.025	0.564
ALMI									−0.078	0.159
ECW/ICW										
ECW/ICW	0.366	<0.001	0.044	0.424	0.05	0.328	0.046	0.374	0.035	0.501
Physical activity							−0.028	0.52	−0.025	0.559
ALMI									−0.08	0.148
BCM/FFM										
ECW/ICW	−0.359	<0.001	−0.036	0.508	−0.043	0.395	−0.039	0.446	−0.028	0.589
Physical activity							−0.029	0.509	−0.026	0.549
ALMI									−0.081	0.143

*Note*: Crude model, no covariates; model 1, sex and age; model 2, model 1 + height, smoking status, history of hypertension, cardiovascular diseases, diabetes, dyslipidemia, and renal failure; model 3, model 2 + objectively measured physical activity level; model 4, model 3 + appendicular lean mass index.

Abbreviations: ALMI, appendicular lean mass index; BCM/FFM, body cell mass‐to‐free fat mass ratio; ECW/ICW, extracellular‐to‐intracellular water ratio; PhA, phase angle.

## 4 DISCUSSION

In this study, our findings revealed that poor PhA, higher ECW/ICW ratio, and lower BCM/FFM were significantly associated with higher cfPWV after adjusting for sex, age, height, smoking status, objectively measured physical activity levels, history of hypertension, CVD, diabetes, dyslipidemia, renal failure, and ALMI. Notably, these associations were independent of ALMI and objectively measured physical activity levels. Previous studies have reported that worsening PhA, higher ECW/ICW ratio, and lower BCM/FFM are linked to functional decline, sarcopenia, falls, and disabilities (Di Vincenzo et al., 2021; Iwasaka et al., 2023; Lukaski & Talluri, 2023; Norman et al., 2023; Uemura et al., 2020; Yamada et al., 2021, 2023). Moreover, poor PhA and higher ECW/ICW ratios have been associated with a higher risk of mortality (Garlini et al., 2019; Iwasaka et al., 2024; Wilhelm‐Leen et al., 2014). Worsening muscle quality, characterized by lower PhA, higher ECW/ICW, and lower BCM/FFM, may lead to mortality by promoting functional decline, sarcopenia, falls, and disability, ultimately leading to bedridden and inactive lifestyles. In addition, our findings provide further evidence that arterial stiffness may serve as a pathway linking poor muscle quality to mortality. cfPWV is a key measure of arterial stiffness, which is an important variable in assessing vascular aging (Marshall et al., 2024). Arterial stiffness, as measured by PWV, exists independently of other cardiovascular diseases and is a significant factor reflecting vascular aging and predicts CVD (Vlachopoulos et al., 2019). Therefore, arterial stiffness and CVD may be important mechanisms explaining the relationship between PhA, ECW/ICW ratio, BCM/FFM, and mortality.

Recent studies have examined the relationship between PhA and CVD. A lower PhA was associated with a higher Framingham risk score, which estimates the risk of CVD, and a point score based on the categorical variables such as age, total cholesterol, HDL‐cholesterol, systolic blood pressure, smoking, and diabetes (Raji et al., 2023) Furthermore, a lower PhA value was associated with a higher incidence of CVD in women over a 24‐year period, even after adjusting for body weight, height, age, smoking status, alcohol intake, physical activity, and education level (Langer et al., 2021). A previous study investigated the association between changes in PhA over a 6‐year period and the risk of all‐cause mortality and incidence of CVD over an 18‐year follow‐up. The findings revealed that individuals who exhibited a decline in PhA of more than 1° had a significantly increased risk of all‐cause mortality and CVD (Langer et al., 2023). Similarly, a study of healthy Danish adults revealed that excess extracellular water related to body water (ECW/TBW), as assessed by BIS, was significantly associated with the incidence of nonfatal or fatal CVD over a median follow‐up of 13.5 years (Knudsen et al., 2014). Although these studies highlight important associations between muscle quality, water distribution, and the incidence of CVD, the underlying mechanisms remain unclear. cfPWV, a measure of arterial stiffness and compliance, is an important predictor of the age‐related development of CVD, as well as the risk of CVD and cardiovascular mortality (Marshall et al., 2024; Vlachopoulos et al., 2019). The most recent study (Tsilingiris et al., 2024) reported that PhA was negatively correlated with cfPWV and other biomarkers associated with adverse cardiovascular profiles, such as renal resistive index, N‐terminal brain natriuretic peptide, and high‐sensitivity troponin‐T levels, in individuals with and without diabetes mellitus. These associations remained statistically significant after adjusting for multiple confounders, including those known to affect PhA measurements, established CVD, and cardiovascular risk factors. Our findings further support the relationship between PhA and cfPWV. Additionally, this study contributes new evidence showing that a higher ECW/ICW ratio and lower BCM/FFM are further significantly associated with higher cfPWV.

Several reasons may exist for the association between PhA, ECW/ICW ratio, and FFM, and cfPWV, even adjusting for multiple covariates such as sex, age, physical activity, and ALMI. Notably, significant associations were observed even after adjusting for objectively measured physical activity levels, a key behavioral confounder. Furthermore, PhA, the ECW/ICW ratio, and the BCM/FFM ratio remained significantly associated with cfPWV independently of ALMI. These findings suggest that these indicators may be associated with vascular function and CVD through mechanisms that are distinct from those of muscle mass.

PhA reflects cell integrity, size, hydration status, and cell death. Therefore, lower PhA levels are associated with reduced cell structure, increased cell death, and overall poorer cell function and health (Nescolarde et al., 2023). PhA is further associated with oxidative stress, cell damage, and inflammation (Bennett et al., 2018; da Silva et al., 2022). These chronic conditions can impair endothelial and smooth muscle function while also promoting vascular fibrosis, calcification, and remodeling. Collecting these changes contributes to elevated PWV, increased arterial stiffness, and the risk of CVD. The ECW/ICW ratio represents the distributions of ECW and ICW. Water plays a key role in metabolism, transport mechanisms, and the maintenance of structural frameworks within the cell (Lorenzo et al., 2019). Excessive accumulation of ECW can induce hyperosmolarity, leading to oxidative stress, impaired cellular function, apoptosis, cell death, and the development of many diseases (Brocker et al., 2012; Lorenzo et al., 2019). In contrast, higher levels of ICW and increased cell volume promote cellular metabolism, activate intracellular signaling pathways, and enhance cellular functions (Lang et al., 1998). Therefore, deterioration in basic metabolism, transport, cellular metabolism, intracellular signaling, and cellular functions negatively affects vascular compliance and remodeling. Furthermore, in hemodialysis patients, an association has been observed between an increased ECW/ICW ratio, higher PWV, and a larger common carotid artery diameter (Kim et al., 2017; Lin et al., 2003). A previous study reported that these changes are not due to acute exposure to high ECW/ICW but rather reflect chronic elevation of extracellular water, which causes changes in forces (shear stress) on the vessel, resulting in wall thickening and a reduction in inner diameter (Lin et al., 2003).

Conversely, a reverse association should be further considered. Previous studies reported that increased arterial stiffness could precede the loss of muscle tissue, as calcified and stiffened blood vessels may hinder the diffusion of oxygen and nutrients to muscles through vascular remodeling. This impairment in nutrient supply can lead to muscle atrophy, cell membrane damage, and ultimately, cell death (Harada et al., 2020; Nilsson et al., 2013). Additionally, several pathways, including insulin resistance, oxidative stress, and inflammation, are common in muscle loss and increased arterial stiffness, offering insights into their underlying mechanisms (Rodríguez et al., 2017). Therefore, an interactive association may exist between poor PhA, higher ECW/ICW ratio, lower BCM/FFM, and increased arterial stiffness. To clarify these underlying biological mechanisms, future studies should incorporate blood biomarkers, more precise physiological measurements, and longitudinal research designs.

Our findings are consistent with those of previous systematic reviews and meta‐analyses, collating findings from observational studies that collectively demonstrate that low muscle tissue is consistently associated with increased arterial stiffness and PWV (Rodríguez et al., 2017). Since electrical properties derived from muscle quality, including PhA, ECW/ICW ratio, and BCMFFM, were strongly related to muscle tissue mass, the loss of muscle tissue mass could partly explain the association between muscle quality and arterial stiffness. However, this relationship remained significant even after adjusting for ALMI, suggesting that muscle quality, cell membrane condition, and water distribution were independently associated with arterial stiffness. These findings underscore the importance of focusing on muscle quality and mass. Additionally, in our study, we employed an objective measure of physical activity level as a covariate in the statistical analysis. Physical activity level may significantly confound the association between body composition and arterial stiffness (Ashor et al., 2014). The use of objectively measured physical activity levels as adjustment variables may allow a suitable assessment of the association between body composition and arterial stiffness.

However, although significant associations were observed in the crude model, no significant associations were found between baPWV and BIS‐derived muscle quality indicators after adjusting for potential covariates, such as sex, age, several chronic disease conditions, objectively measured physical activity, and ALMI. The associations observed in the crude model may have been influenced by confounding factors, such as age and sex.

Several studies have reported associations between baPWV and sarcopenia or cardiovascular risk (Marshall et al., 2024; Piotrowicz et al., 2022), yet baPWV is widely used in large‐scale studies and primary care settings due to baPWV practicality and predictive value. However, unlike the direct ECG‐gated cfPWV measurement, baPWV assessed using a cuff‐based method is not considered the gold standard for evaluating arterial stiffness (Laurent et al., 2006; Piotrowicz et al., 2022). The cuff‐based baPWV method has been criticized for being sensitive to age and blood pressure levels as well as for reflecting a composite value derived from multiple arterial segments, including large elastic arteries, such as the descending aorta and stiffer peripheral muscular arteries. In peripheral arteries, wave reflection is more prominent, which introduces noise that complicates waveform analysis (Budoff et al., 2022; Lu et al., 2017; Piotrowicz et al., 2022; Yu et al., 2008).

In contrast, cfPWV captures a shorter arterial segment limited to central elastic arteries and directly measures the distance between the carotid and femoral arteries. This approach minimizes error due to interindividual anatomical differences. cfPWV is widely considered a superior predictor of cardiovascular outcomes and a more robust indicator of central arterial stiffness. Furthermore, the mechanisms regulating arterial stiffness differ between cfPWV and baPWV. Although cfPWV mainly reflects structural remodeling of the arterial wall, baPWV is more influenced by short‐term physiological responses, such as sympathetic activity and local vascular tone. These differences suggest that, although baPWV may be suitable as a screening tool, cfPWV is more appropriate as a precise index for mechanistic investigations.

Conversely, although an association was observed in the unadjusted model, we found no association between muscle quality and baPWV after adjusting for potential covariates such as sex, age, physical activity, and ALMI. The association between baPWV and PhA, ECW/ICW ratio, and BCM/FFM may have been influenced by confounding factors, such as sex and age. Although several studies have suggested that baPWV is associated with sarcopenia (Piotrowicz et al., 2022), baPWV measured using the cuff‐based method is not the gold standard for direct ECG‐gated cfPWV measurement (Laurent et al., 2006; Piotrowicz et al., 2022). The cuff‐based baPWV method has been criticized for having strong dependence on age and blood pressure levels, as well as for including multiple arterial segments into a single composite value. This includes large elastic arteries, such as the descending aorta and stiffer peripheral arteries—namely, large conduit arteries (mainly the aorta) and muscular arteries (brachial and tibial) (Budoff et al., 2022; Lu et al., 2017; Piotrowicz et al., 2022; Yu et al., 2008). In this study, muscle quality, including PhA, ECW/ICW ratio, and BCM/FFM, was not associated with arterial stiffness in peripheral arteries. Further research comparing baPWV and cfPWV in relation to sarcopenia, muscle mass, and muscle quality is needed to clarify the reasons for these discrepancies. Nevertheless, the baPWV method has been widely used in numerous studies and clinical settings due to baPWV practicality and ease of implementation.

However, as baPWV measurements reflect not only the aorta but also the muscular arteries, limitations in assessing arteriosclerosis in the central artery, which is important for estimating the risk of CVD, exist. In our study, by measuring the baPWV and cfPWV, we were able to assess arteriosclerosis in the central artery and compare the findings with those of previous studies. Nonetheless, this study had some limitations. First, a key limitation of this study is that it relies on BIS to estimate body composition and water distribution. Although BIS is a widely used, noninvasive technique, BIS accuracy depends on assumptions that may not hold for older adults or individuals with comorbidities. For instance, BIS employs fixed resistivity constants for intracellular and extracellular compartments; however, these values may fluctuate due to age‐related changes in tissue composition or disease‐related alterations. Moreover, using height and weight to approximate body geometry and density may also introduce errors, especially in older adults with atypical body proportions. BIS‐derived estimates can further differ from those obtained through reference methods such as tracer dilution, raising concerns about measurement validity. Additionally, although commonly believed to be unable to penetrate cell membranes, low‐frequency current may partially traverse muscle fibers, depending on the direction and orientation of the current. This variability could potentially influence impedance readings and their physiological interpretation (Ward & Brantlov, 2023). Second, the study was conducted with a cross‐sectional design, which limits our ability to infer a causal relationship between muscle quality and PWV. Further longitudinal and interventional studies are required to confirm this association. Third, as the participants of our study were Japanese individuals residing in two specific districts who voluntarily participated, the generalizability of the findings may be limited due to potential sampling bias. Additional research is necessary to determine whether these findings can be generalized to other populations. Fourth, the BIS measurements used in this study were obtained with the ImpediMed SFB7 device. We acknowledge potential inter‐device variability as a limitation. However, given the likely consistency of measurement bias direction, we consider its impact on the associations between BIS‐derived variables and PWV to be minimal.

In conclusion, BIS‐derived parameters reflecting muscle cell mass, cell membrane condition, and water distribution—namely PhA, ECW/ICW, and BCM/FFM—were significantly associated with cfPWV, even after adjusting for sex, age, height, smoking status, objectively measured physical activity, history of hypertension, CVD, diabetes, dyslipidemia, renal failure, and ALMI. Notably, these relationships were independent of ALMI and objectively measured physical activity levels. These findings suggest that targeting these parameters may help maintain arterial stiffness and vascular health. Moreover, they provide new evidence that may partly explain how a decline in these BIS‐assessed parameters contributes to mortality due to poor vascular conditions or CVD. This underscores the importance of prioritizing BIS parameters in strategies for health promotion and disease prevention.

## AUTHOR CONTRIBUTIONS

Yujiro Asano: conceptualization, methodology, software, formal analysis, data curation, writing—original draft, writing—review and editing, and funding acquisition. Motohiko Miyachi: data curation, investigation, conceptualization, supervision, writing, review and editing. Hinako Nanri: Conceptualization, methodology, formal analysis, writing–review and editing, supervision, project administration, and funding acquisition. Haruka Murakami: data curation, investigation, conceptualization, project administration, writing, review, and editing. Yuko Gando: data curation, investigation, writing, review, and editing. Takashi Nakagata: data curation, investigation, writing, review, and editing. Tsukasa Yoshida: data curation, investigation, writing–review and editing. Tomohiro Okura: writing, review, editing, and supervision. Yosuke Yamada: conceptualization, methodology, investigation, validation, data curation, writing—review and editing, supervision, project administration, and funding acquisition.

## FUNDING INFORMATION

This study was supported by grants from Funder One, Health and Labour Sciences Research Grant: 200825016B and 201222028B (to MM).

## CONFLICT OF INTEREST STATEMENT

The authors declare no conflicts of interest.

## ETHICS STATEMENT

The study was approved by the Institutional Review Board of the National Institute of Biomedical Innovation, Health and Nutrition (No. KENEI‐102), and the study adhered to the principles outlined in the Declaration of Helsinki. Written informed consent was obtained from all participants before their participation.

## Data Availability

The data are not publicly available due to privacy and ethical restrictions. The data supporting the findings of this study are available from the corresponding author upon request.
